# Supplementary material for: Impact of insular landscape features on the population genetics of a threatened climbing palm, Korthalsia rogersii Becc., endemic to the Andaman Islands
Source: PeerJ. 2025 Nov 10;13:e20265. doi: 10.7717/peerj.20265 (PMC12614096; doi:10.7717/peerj.20265)
Supplement: Supplemental Information 1 [file peerj-13-20265-s001.docx]

| Table S1. Microsatellite loci genotyped and their corresponding primer specifications. | | | | |
| --- | --- | --- | --- | --- |
| Primer ID | Sequence (5’-3’) | Repeat | Annealing temperature | Observed Allele Size range |
| KleSSR16 | F- GTGGATCGGACGGGATTTCT | (TTA)22 | 61.2 | 147 to 246 |
|  | R- GTTAGGTGGGCTTGGTAGGG |  |  |  |
| KleSSR18 | F- CATGGGATGCGATGGGATGG | (GAT)9 | 63.1 | 188 to 112 |
|  | R- CCCAGGCAGACAGCTGTATT |  |  |  |
| KleSSR23 | F- AATCCCTGCGGCGAAGAATT | (CTC)10 | 66.4 | 182 to 200 |
|  | R- CCGGAATCTGGACCAGTGAC |  |  |  |
| KLeSSR30 | F- GTACTCCGTTCCATGGGCC | (CTC)10 | 66.4 | 184 to 220 |
|  | R - TCTCCTCGTCCTCAATTGCG |  |  |  |
| KleSSR03 | F - TGGTACCATGCTGTTCACCA | (AT)15 | 60.9 | 175 to 199 |
|  | R- TGTTCATGAATAGTCCGGCC |  |  |  |
| KleSSR13 | F- TTGGCATCCGTCCATCACTC | (TTA)20 | 59.6 | 162 to 248 |
|  | R- GCCCAGTGGTTTTAGTCGGT |  |  |  |
| KlEST24 | F- GCCCAAAGTGATGCCATGAA | (CTC)7 | 62.7 | 211 to 220 |
|  | R- CTCTTCTTTGATGACGGCGG |  |  |  |
| All the PCR amplifications were carried out in 20 μL reaction volume containing 5-10 ng DNA, 10x Taq buffer with 1.5 mM MgCl2, 200 μM dNTPs, 10 pm of each primer and 2 U Taq DNA polymerase. Standardized PCR conditions were initial denaturation at 95°C for 5 min, followed by 34 cycles at 94°C for 45 s, standardized annealing temperatures for each primer set for 60 s (Table S1), an extension period at 72°C for 45 s and final extension at 72°C for 10 min. | | | | |

| Table S2. HWE globally - across all 98 individuals sampled and genotyped | | | | |
| --- | --- | --- | --- | --- |
|  | chi^2 | df | Pr(chi^2>) | Pr.exact |
| Kle16 | 232.862 | 231 | 0.453 | 0.489 |
| Kle18 | 20.861 | 21 | 0.467 | 0.04 |
| Kle23 | 102.988 | 21 | 0 | 0 |
| Kle30 | 111.424 | 55 | 0 | 0.001 |
| Kle3 | 103.491 | 36 | 0 | 0.001 |
| Kle13 | 361.337 | 190 | 0 | 0.196 |
| Klest24 | 48.358 | 6 | 0 | 0.008 |
| Chisq (Chi-squared test), MC (Monte Carlo permutation test) | | | | |

| Table S2b. Proportion of populations out of HWE after false discovery rate (fdr) correction | | | | |
| --- | --- | --- | --- | --- |
| Population | Chisq | MC | Chisq.fdr | MC.fdr |
| Interview_island | 0.428571 | 0.142857 | 0.285714 | 0 |
| Radhanagar | 0 | 0 | 0 | 0 |
| Betapur | 0.142857 | 0.285714 | 0 | 0 |
| Bakultala | 0.142857 | 0.142857 | 0 | 0 |
| Baratang | 0.142857 | 0.142857 | 0 | 0 |
| Havelock | 0.142857 | 0.142857 | 0 | 0 |
| ChidiyaTapu | 0.428571 | 0.285714 | 0.285714 | 0 |
| *Chisq* (Chi-squared test), *MC* (Monte Carlo permutation test), Chisq.fdr and MC.fdr are Chiq and MC after fdr correction | | | | |

| Table S2a. Proportion of loci out of HWE across populations | | |
| --- | --- | --- |
|  | Chisq | MC |
| Kle16 | 0 | 0 |
| Kle18 | 0 | 0 |
| Kle23 | 0.285714 | 0.571429 |
| Kle30 | 0.428571 | 0.285714 |
| Kle3 | 0.428571 | 0.142857 |
| Kle13 | 0.142857 | 0 |
| Klest24 | 0.142857 | 0 |
| Chisq (Chi-squared test), MC (Monte Carlo permutation test) | | |

| Table S3. Summary of bottleneck analysis results for seven populations of *Korthalsia rogersii* under the two-phase mutation model (TPM) and stepwise mutation model (SMM). | | | | | | |
| --- | --- | --- | --- | --- | --- | --- |
| Population | mean_k | mean_He | p_W_1t_TPM | p_W_2t_TPM | p_W_1t_SMM | p_W_2t_SMM |
| Radhanagar | 4.43 | 0.6178 | 0.9727 | 0.0781 | 0.9805 | 0.0547 |
| Interview_island | 6 | 0.6593 | 0.9805 | 0.0547 | 0.9805 | 0.0547 |
| Betapur | 4.57 | 0.6243 | 0.5938 | 0.9375 | 0.5938 | 0.9375 |
| Bakultala | 4.57 | 0.5780 | 0.7656 | 0.5781 | 0.7656 | 0.5781 |
| Baratang | 4.71 | 0.6304 | 0.6563 | 0.8125 | 0.7656 | 0.5781 |
| Havelock | 5.29 | 0.6257 | 0.9609 | 0.1094 | 0.9609 | 0.1094 |
| ChidiyaTapu | 7 | 0.6514 | 0.9961 | 0.0156 | 1.0000 | 0.0078 |
| Mean_k: Average number of alleles per locus, Mean_He: Average expected heterozygosity across loci, p (Wilcoxon, 1-tailed, TPM/SMM): p-value from one-tailed Wilcoxon signed-rank test for heterozygote excess under the TPM or SMM models. Values below 0.05 suggest potential recent bottlenecks, p (Wilcoxon, 2-tailed, TPM/SMM): p-value from two-tailed Wilcoxon signed-rank test under the TPM or SMM. Values indicate deviation (either excess or deficit) from mutation-drift equilibrium. | | | | | | |
