# Supplementary figures and images for: Impact of insular landscape features on the population genetics of a threatened climbing palm, Korthalsia rogersii Becc., endemic to the Andaman Islands
Source: PeerJ. 2025 Nov 10;13:e20265. doi: 10.7717/peerj.20265 (PMC12614096; doi:10.7717/peerj.20265)

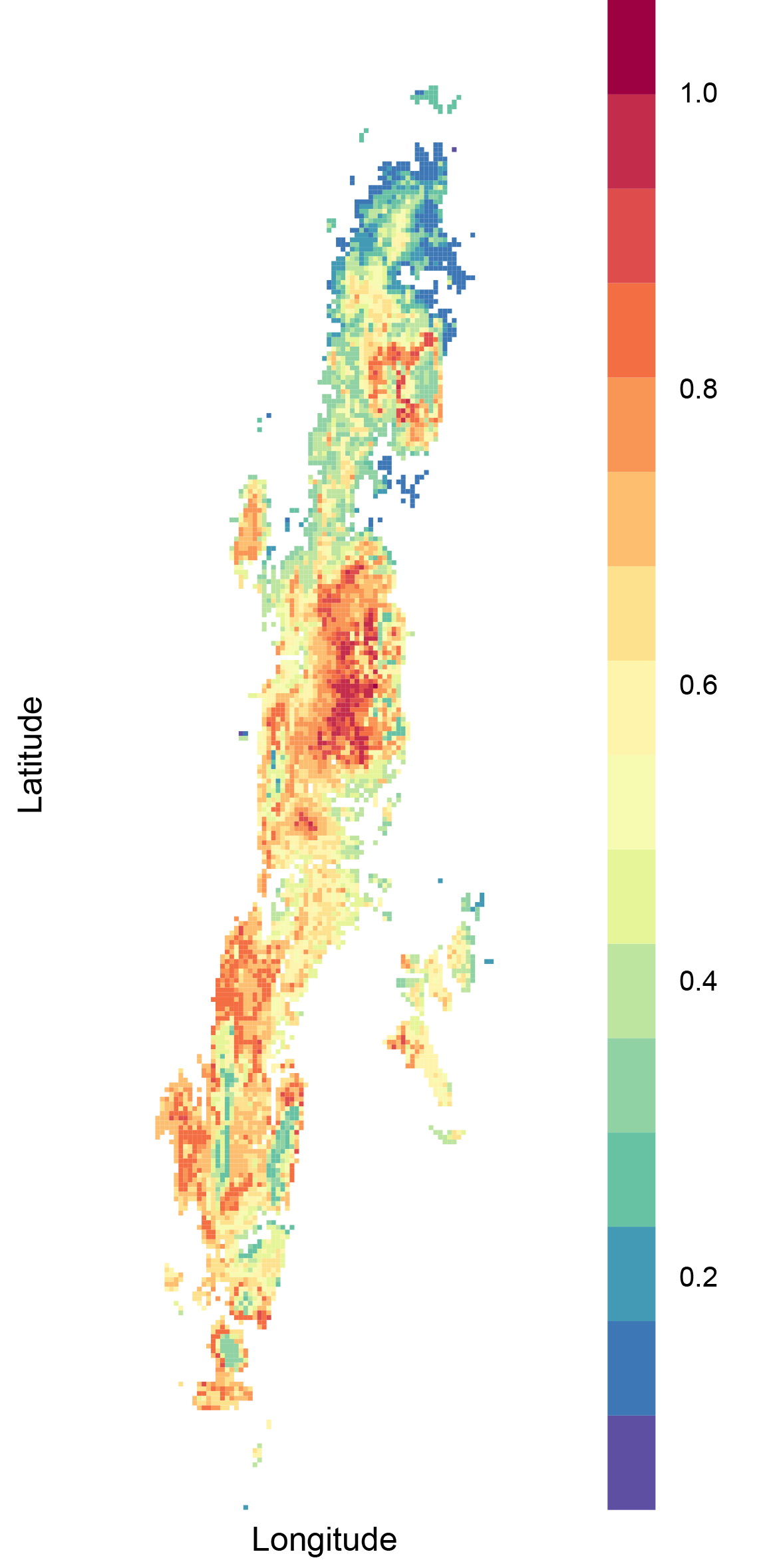

Supplement: Supplemental Information 2 [file peerj-13-20265-s002.png]

Number of alleles

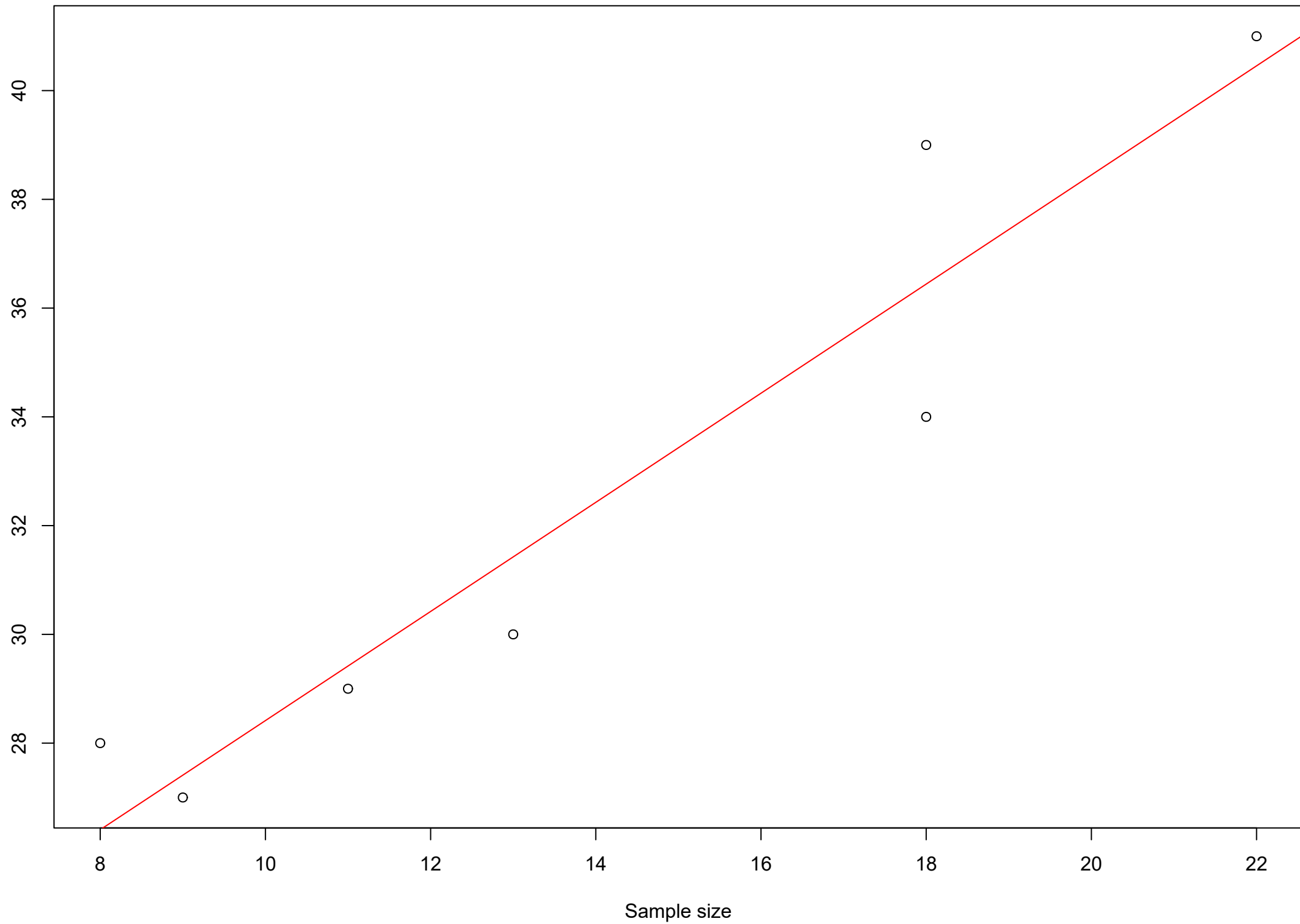

Supplement: Supplemental Information 3 [file peerj-13-20265-s003.pdf]

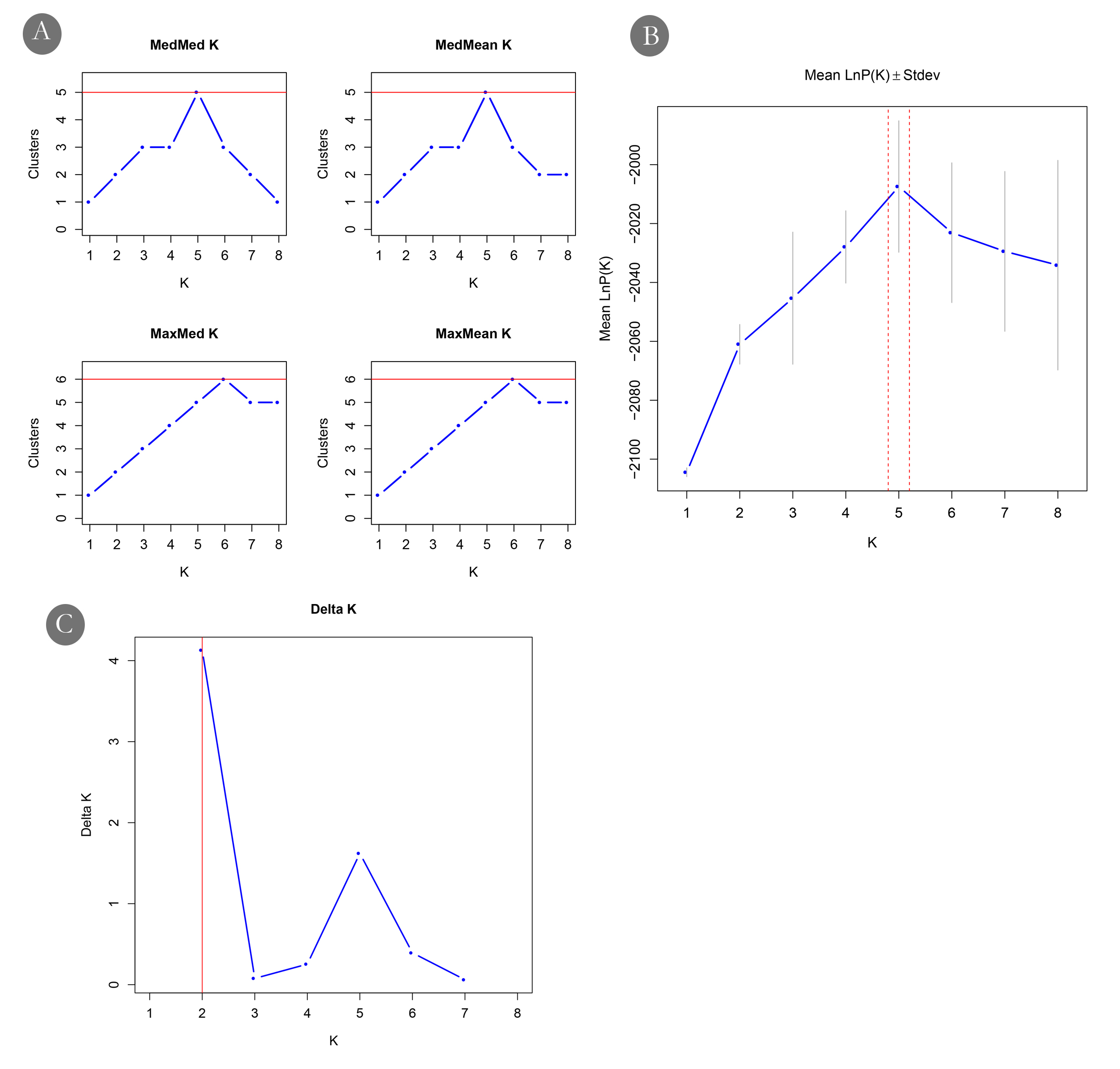

Supplement: Supplemental Information 4 [file peerj-13-20265-s004.png]
